# Clinical Outcomes Following Discordance Between Fractional Flow Reserve and Instantaneous Wave-Free Ratio in Deferred Coronary Lesions: A Systematic Review and Meta-Analysis

**DOI:** 10.31083/RCM44868

**Published:** 2025-11-21

**Authors:** Spyridon Graidis, Filippos Timpilis, Georgia Xygka, Asimenia Katsea, Antonios Karanasos, Grigorios Tsigkas, Athanasios Moulias, Virginia Mplani, Periklis Davlouros, Michail Papafaklis

**Affiliations:** ^1^Cardiology Division, University Hospital of Patras, 26504 Rio, Greece; ^2^Faculty of Medicine, University of Patras, 26504 Rio, Greece; ^3^Intensive Care Unit, University Hospital of Patras, 26504 Rio, Greece

**Keywords:** coronary artery disease, coronary stenoses, clinical decision, fractional flow reserve, instantaneous wave-free ratio, mortality, percutaneous coronary intervention, revascularization

## Abstract

**Background:**

Current guidelines recommend the use of either fractional flow reserve (FFR) or instantaneous wave-free ratio (iFR) for assessing intermediate coronary stenoses. However, FFR/iFR discordance occurs in approximately 20% of cases. This systematic review and meta-analysis aimed to investigate whether deferring lesions with discordant FFR/iFR classification is associated with worse prognosis compared to those with negative concordant results (FFR–/iFR–).

**Methods:**

A systematic search was conducted in literature repositories to identify all studies that compared the clinical prognosis of deferred lesions with discordant and concordant FFR/iFR results. The primary endpoint was a composite clinical outcome of the individual secondary endpoints (death, myocardial infarction, and revascularization).

**Results:**

Three eligible observational studies (1735 deferred vessels) were included in the meta-analysis. Overall, deferred lesions with FFR/iFR discordance presented numerically higher event rates for all primary and secondary endpoints compared to deferred lesions with negative concordance; however, none reached statistical significance. Deferred lesions with FFR–/iFR+ discordance were significantly associated with an increased risk of death (odds ratio [OR]: 3.19; *p* = 0.049), while deferred lesions with FFR+/iFR– discordance were associated with a greater risk of revascularization compared to deferred lesions with negative concordance (OR: 3.24; *p* = 0.01).

**Conclusions:**

Compared to deferred lesions with negative concordant results, deferred lesions with discordant FFR/iFR results were overall not significantly associated with worse clinical outcomes; however, there was a significantly greater risk of death for deferred lesions specifically with FFR–/iFR+ discordance, and an increased risk of revascularization for deferred lesions with FFR+/iFR– discordance. Further dedicated trials are needed to improve guidance in clinical decision-making.

**The PROSPERO Registration:**

CRD420251135424, https://www.crd.york.ac.uk/PROSPERO/view/CRD420251135424.

## 1. Introduction

The diagnostic performance of elective invasive coronary angiography is limited because visual assessment during coronary angiography cannot precisely evaluate the functional significance of intermediate stenoses [1, 2]. A visual-functional mismatch is evident in 42–57% of non-left main lesions and 35–55% of left main lesions, while a reverse mismatch is apparent in 14–16% of non-left main lesions and 14–40% of left main lesions [1, 2].

Fractional flow reserve (FFR) is the most commonly used physiologic index to assess the functional importance of intermediate coronary lesions. Landmark trials have demonstrated that deferral of FFR-based non-significant lesions is safe [3], and FFR-guided percutaneous coronary intervention (PCI) leads to improved clinical outcomes compared to optimal medical treatment [4]. More recently, the instantaneous wave-free ratio (iFR), a non-hyperemic pressure ratio (NHPR) not requiring the use of a hyperemic agent such as adenosine, has been developed. DEFINE-FLAIR and iFR SWEDEHEART are two landmark randomized control trials comparing FFR- vs. iFR-guided PCI approaches, showing that iFR-guided revascularization is non-inferior to FFR-guided revascularization [5, 6]. Both the European Society of Cardiology (ESC) and American College of Cardiology (ACC) guidelines on chronic coronary syndromes have given a class I indication (level of evidence A) for the equivalent use of FFR or iFR for the functional assessment of angiographically intermediate coronary lesions before deciding on deferring or proceeding to PCI [7, 8]. Although other NHPRs (e.g., resting full-cycle ratio [RFR], diastolic hyperemia-free ratio [DFR], and diastolic pressure ratio [DPR]) have also been lately proposed and show very high correlation to iFR results, they have not been tested for outcomes in a randomized clinical setting; thus, they have been given a much lower class indication (IIb; level of evidence C) and are not recommended equivalently to FFR or iFR [7].

Despite the equivalent recommendation for FFR and iFR, FFR/iFR discordance occurs in approximately 20% of functionally assessed lesions, with the incidence of discordance ranging from 8% to 40% according to the cut-off values used in each study [9]. Several factors have been identified as predictors of FFR/iFR discordance, including sex, age, insulin-treated diabetes mellitus, chronic kidney disease, angiographic lesion characteristics, elevated left ventricular end-diastolic pressure, and atrial fibrillation [10, 11, 12, 13, 14, 15, 16].

It has not been established whether FFR/iFR discordance has clinical implications and what the appropriate treatment strategy should be for such discordant lesions. Evidence remains unclear, given that some studies showed similar mid-term clinical outcomes between the discordant and negative concordant groups, and other studies showed that the former may be associated with worse clinical prognosis in the long-term [17, 18]. Until now, no meta-analytic data focusing on FFR/iFR discordance have been presented.

The aim of our study was to conduct a systematic review and meta-analysis that compares the clinical outcomes between deferred lesions with discordant FFR/iFR results (i.e., FFR+/iFR– or FFR–/iFR+) and deferred lesions with negative concordance (FFR–/iFR–).

## 2. Materials and Methods

The systematic review and meta-analysis followed the Preferred Reporting Items for Systematic Reviews and Meta-Analyses (PRISMA) guidelines [19, 20], and were registered in the PROSPERO database (CRD420251135424).

### 2.1 Search Strategy

We screened PubMed, Cochrane (CENTRAL), Web of Science, and Epistemonikos.org for all eligible studies with both FFR and iFR measurements, comparing the clinical outcomes of FFR/iFR concordance and discordance. The following query string was used: (“FFR” OR “fractional flow reserve”) AND (“iFR” OR “instantaneous wave-free ratio”). Additionally, we used the “snowball technique” to identify other relevant studies. Finally, we searched grey literature by scanning abstracts of major cardiology meetings and the ClinicalTrials.gov database. No study design or language restrictions were placed, ensuring a comprehensive search strategy.

### 2.2 Eligibility Criteria

The meta-analysis included studies that assessed the clinical outcomes between deferred lesions with negative concordance (i.e., FFR–/iFR–) and deferred lesions with discordant FFR/iFR results (either FFR+/iFR– or FFR–/iFR+); FFR+ corresponds to FFR ≤0.80 with FFR– to FFR >0.80, and iFR+ corresponds to iFR ≤0.89 with iFR– to iFR >0.89. Given our specific focus on FFR and iFR, i.e., the NHPR which is recommended equivalently to FFR by the American and European guidelines, any study which presented NHPR results without separately providing outcomes for iFR was not included. The primary endpoint was a composite clinical outcome consisting of death (either cardiac or all-cause death), myocardial infarction, and revascularization (either target-lesion revascularization or revascularization, irrespective of lesion location). The secondary endpoints were death, myocardial infarction, and revascularization.

### 2.3 Study Selection and Data Extraction

After data retrieval, all studies were imported into the reference management software (EndNote 20.5, Clarivate Analytics, Philadelphia, PA, USA). Then, the deduplication process occurred, and the abstracts were initially screened for relevance to the research question. The remaining studies were assessed according to the inclusion criteria. Two independent reviewers were responsible for the whole study selection process, and a third senior reviewer resolved any disparities by reaching a consensus.

Then, the data extraction process took place. Two independent reviewers were responsible for data extraction, and any discrepancies were resolved by a third senior reviewer who reached a consensus to ensure the reliability of the data extraction process.

Qualitative outcomes were measured as frequencies and percentages. The full text and appendices were reviewed in accordance with the Cochrane guidelines for systematic reviews and meta-analyses [21].

### 2.4 Risk of Bias Assessment

Two independent reviewers used the appropriate tools (the revised Robins-I tool for observational studies and the ROB2 tool for randomized trials), as proposed by the Cochrane Collaboration guidelines, to assess the risk of bias regarding the composite clinical outcome [21, 22, 23]. The results were visualized using the “robvis” tool (accessible at https://mcguinlu.shinyapps.io/robvis/).

### 2.5 Data Analysis and Statistics

The primary analysis consisted of a fixed-effects model meta-analysis comparing deferred lesions with discordant FFR/iFR results to deferred lesions with negative concordance. The effect estimates for the clinical outcomes were presented as odds ratios and 95% confidence intervals. Statistical inconsistency was assessed using the Q-test (chi-squared test) and the I^2^ statistic. An I^2^ metric <25% signified low heterogeneity, 25–50% signified moderate, and >50% signified high heterogeneity. In the case of high heterogeneity, a sensitivity analysis (with exclusion of studies with a high risk of bias) was planned. Additionally, a leave-one-out analysis was planned to assess the individual influence of each study on the results of the meta-analysis. Publication bias was evaluated by examining the funnel plot asymmetry of the comparison-adjusted funnel plot and by conducting Egger’s test in cases where more than 10 comparisons were made for the outcome of interest [24, 25].

A secondary analysis consisted of a common effects network meta-analysis. The effect estimates for the clinical outcomes were presented as odds ratios and 95% confidence intervals. Statistical inconsistency was assessed using both a local and global approach. The local approach involved using the node-splitting or SIDE (Separating Indirect from Direct Evidence) method, as per the Cochrane Collaboration guidelines [26]. The global inconsistency assessment was conducted using the Q-test (chi-squared test) and the I^2^ statistic. In the case of high heterogeneity, a sensitivity analysis (with exclusion of studies with a high risk of bias) was planned. The P-metric was utilized to rank treatments. This metric signifies the proportion of certainty that a treatment is more effective than another treatment, taking into account all the treatment options compared [27]. Furthermore, we assessed each study’s impact on the network by measuring the percentage decrease in precision following the removal of the study from the network [28]. Finally, publication bias was evaluated [29].

For the analysis, the cut-off for statistical significance was defined as a *p*-value less than 0.05 and a 95% CI that did not include the unit of measure. The analysis was conducted using the R statistical software (version 4.2.3; R Core Team 2023, R Foundation for Statistical Computing, Vienna, Austria) and the packages “meta”, “metafor”, and “netmeta”.

## 3. Results

**Fig. 1.**
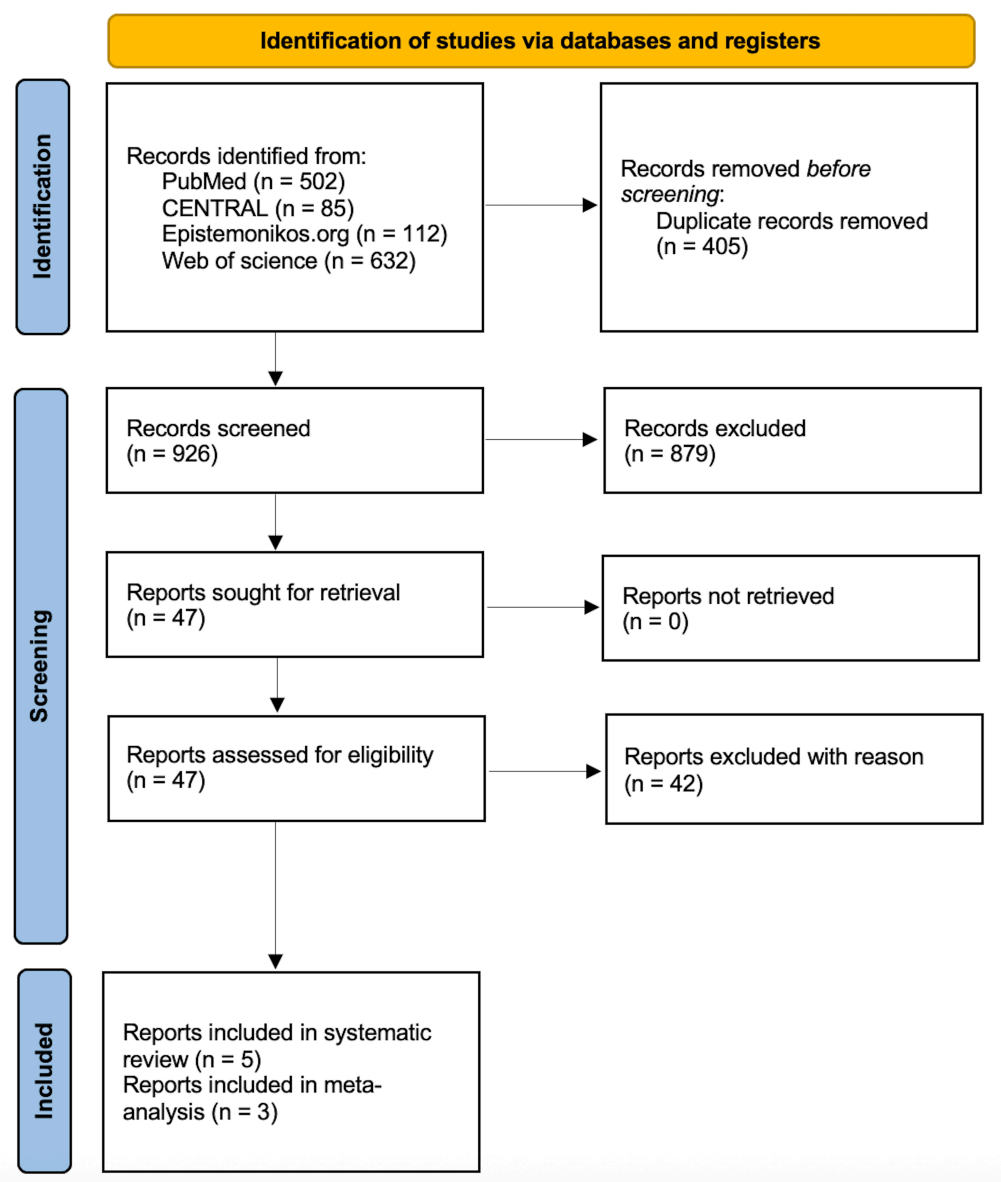
**PRISMA flow chart**. The chart depicts the processes of database searching and study selection. 926 studies were screened, and only 5 reports of 3 observational studies were included in the final systematic review, while only 3 reports were included in the meta-analysis. PRISMA, Preferred Reporting Items for Systematic Reviews and Meta-Analyses.

Following the search strategy, 1331 reports were retrieved. After the deduplication and the initial screening of titles and abstracts for relevance, 47 reports remained. These articles were assessed against the eligibility criteria, and ultimately, only 5 reports of 3 observational studies were included in the analysis [17, 18, 30, 31, 32]. Details about the screening process can be found in Fig. 1.

**Fig. 2.**
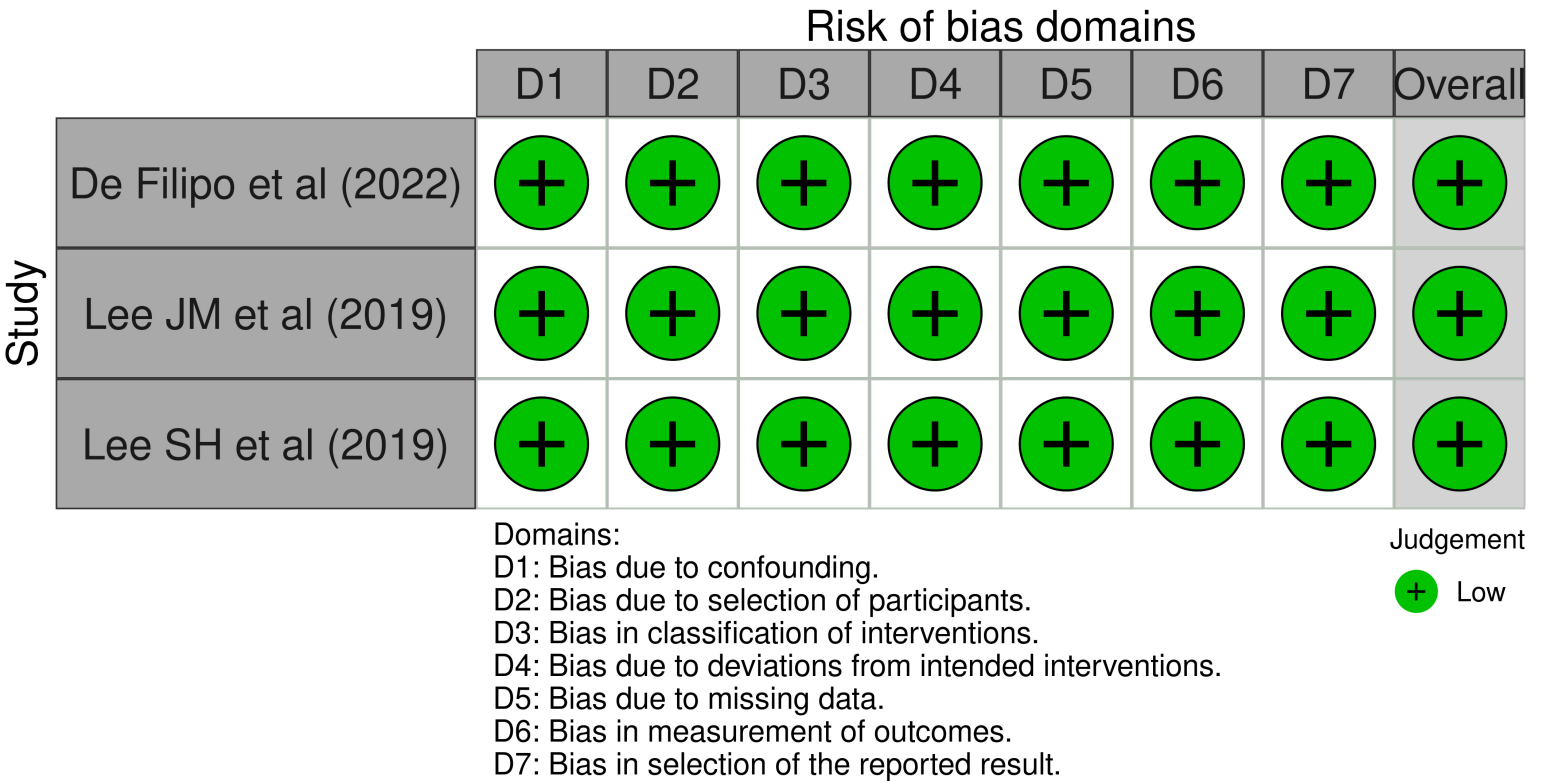
**Risk of bias assessment**. This figure illustrates the results of the risk of bias assessment using the Robins-I tool proposed by the Cochrane Collaboration. Overall, all three studies were assessed as having low risk of bias.

Regarding the risk of bias assessment, all included studies were observational, and were thus evaluated using the Robins-I tool; the evaluation showed a low risk of bias (Fig. 2).

### 3.1 Systematic Review

Firstly, Lee JM *et al*. [18, 30, 31, 33] have published a series of 3 reports that investigated the clinical outcomes of deferred coronary lesions with either discordant or concordant FFR/iFR results, including participants from either the 3V FFR–FRIENDS study or the 3V FFR–FRIENDS study combined with the ^13^N-ammonia positron emission tomography (PET) registry. The 3V FFR–FRIENDS study included adult patients with visually estimated coronary stenosis greater than 30% who underwent FFR/iFR measurement in all three coronary vessels, and its goal was to assess whether a low total sum of FFR in all three coronary vessels (3V FFR) is associated with worse outcomes compared to high FFR [34]. The ^13^N-ammonia PET registry included patients with stenosis in the left anterior descending artery (LAD) who underwent FFR and iFR measurements, and subsequently underwent ^13^N-ammonia PET to assess coronary flow reserve (CFR) and relative flow reserve (RFR) [35]. Their first report, which included a total of 821 deferred coronary vessels from the 3V FFR–FRIENDS study alone, investigated the two-year clinical outcomes of lesions with discordant and concordant results [30]. They demonstrated that deferred lesions with discordant results (either FFR+/iFR– or FFR–/iFR+) were not significantly associated with a greater risk of death, vessel-related ischemia-driven revascularization, vessel-related myocardial infarction, and major adverse cardiac events (MACE) compared to deferred lesions with negative concordant results [30]. Their second report included a larger sample size than the first report; it included a total of 864 deferred vessels from both the 3V FFR–FRIENDS study and the N-ammonia PET registry [31]. In the second report, they concluded that deferred lesions with discordant FFR/iFR results do not have an increased risk of cardiac death, ischemia-driven revascularization, myocardial infarction, or vessel-oriented clinical outcomes (VOCO) at two years of follow-up [31]. Their third report investigated the long-term clinical prognosis of deferred lesions with negative concordant and discordant results compared to revascularized lesions [18]. It included 790 deferred vessels from both the 3V FFR–FRIENDS study and the N-ammonia PET registry [18]. The main difference from the previous report is that the follow-up period was substantially longer (5 years), and that the authors made derivations of all NHPRs and excluded cases with discordant classification among NHPRs in order to present comparative clinical outcomes regarding FFR/NHPR discordance/concordance [18]. Notably, it demonstrated that deferred coronary lesions with discordant FFR/NHPR results had a greater risk of ischemia-driven revascularization and VOCO compared to deferred lesions with negative concordant results; however, this risk was comparable to that of revascularized lesions [18]. For the purpose of our meta-analysis, we decided to include the second report of Lee JM *et al*. [31], because it included a larger population than both the first [30] and third [18] reports. Also, the second report included individual results regarding the different FFR/iFR discordance groups (either FFR+/iFR– or FFR–/iFR+), which could be used for the secondary analysis; this piece of information was not available in the third report with the longer follow-up [18].

The study by De Filippo *et al*. [32] included 160 deferred coronary lesions assessed with FFR and iFR, aiming to identify tailored cut-offs for iFR that predicted a positive FFR and to evaluate the impact of lesion reclassification on MACE. The study showed that deferred lesions with discordant results were not significantly associated with a greater risk of MACE, compared to negative concordant lesions.

Finally, the study by Lee SH *et al*. [17] included a total of 711 deferred vessels with available FFR and iFR data, and showed that lesions with discordant results were not associated with a significantly higher risk of clinical outcomes, including all-cause mortality, revascularization and myocardial infarction, when compared to lesions with negative concordant results. However, deferred lesions with iFR–/FFR+ discordance had a trend of a greater risk of revascularization compared to negative concordant lesions; of note, the revascularization rate in deferred lesions with iFR–/FFR+ discordance was similar to the risk of repeat revascularization of lesions with positive concordance which had been initially treated/revascularized [17].

### 3.2 Primary Analysis

In the primary analysis, a total of 1735 deferred vessels from 3 studies were considered; the overall weighted mean follow-up duration was 2.8 years.

**Fig. 3.**
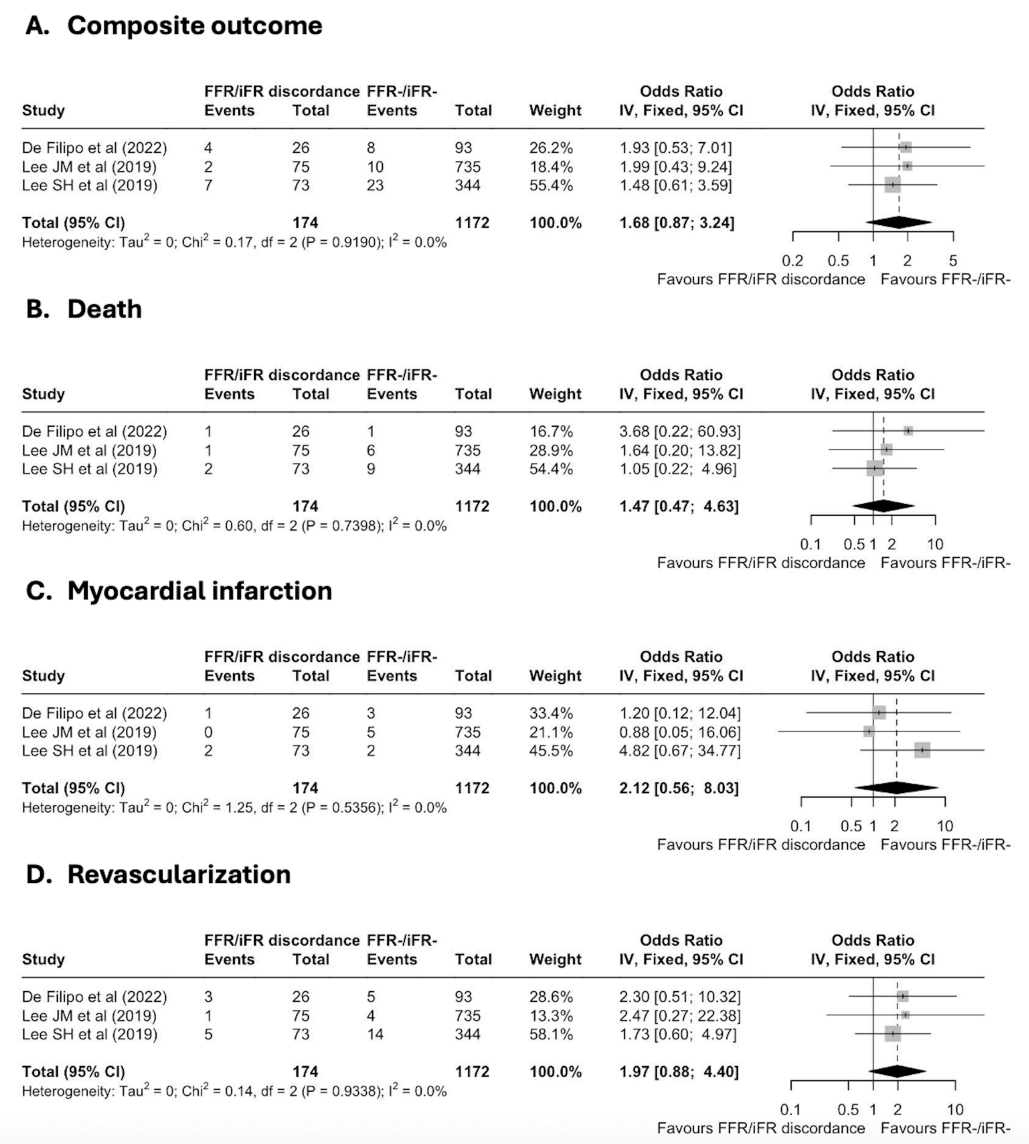
**Forest plots for all the endpoints of the meta-analysis**. (A) Composite clinical outcome (primary endpoint). (B) Death. (C) Myocardial infarction. (D) Revascularization. FFR, fractional flow reserve; IFR, instantaneous wave-free ratio.

Regarding the primary endpoint, there was a numerically higher rate of the composite outcome for deferred lesions with discordant FFR/iFR results compared to the reference group of deferred lesions with negative concordance (FFR–/iFR–) without reaching statistical significance (OR: 1.68, 95% CI 0.87–3.24, *p* = 0.13; Fig. 3A).

Regarding the secondary endpoints, deferred lesions with discordant FFR/iFR results were not significantly associated with an increased risk of death (OR: 1.47, 95% CI 0.47–4.63, *p* = 0.51; Fig. 3B) and myocardial infarction (OR: 2.12, 95% CI 0.56–8.03, *p* = 0.27; Fig. 3C), but presented a trend for a higher revascularization rate (OR: 1.97, 95% CI 0.88–4.4, *p* = 0.098; Fig. 3D) compared to deferred lesions with negative concordance.

Heterogeneity was low (I^2^ = 0%, Q-test *p*-value > 0.5) for all endpoints (Fig. 3) justifying the use of a fixed-effects model. The leave-one-out analysis did not detect the presence of highly influential studies for any outcome (**Supplementary Fig. 1**). Lastly, by visually inspecting the funnel plots for each outcome, we can apparently conclude that there is no noteworthy publication bias (**Supplementary Figs. 2,3,4,5**), although the limitation of the small number of studies makes it difficult to assess publication bias.

### 3.3 Secondary Analysis

**Fig. 4.**
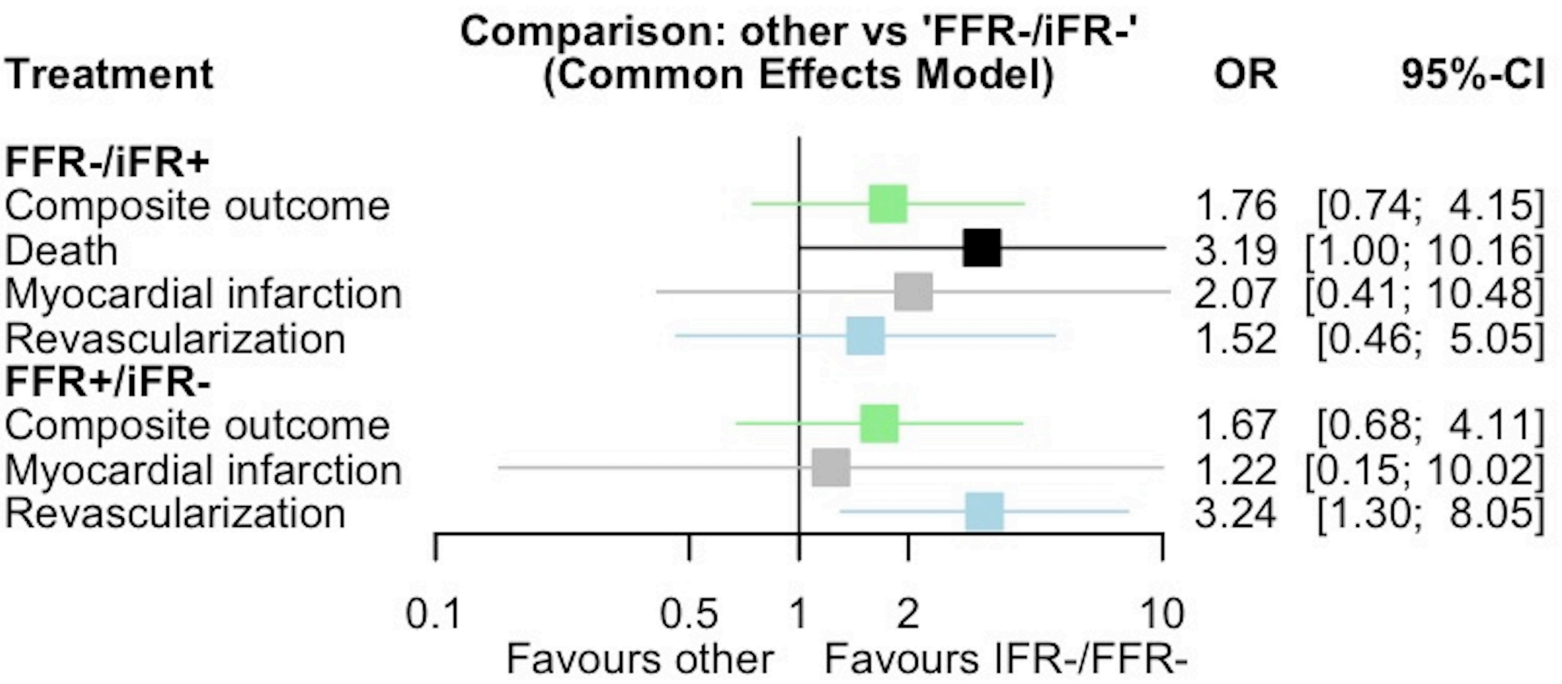
**Forest plot of the network meta-analysis for all endpoints**. Deferred lesions with FFR+/iFR– discordance were significantly associated with increased risk of revascularization compared to deferred lesions with negative concordance (iFR–/FFR–). Deferred lesions with FFR–/iFR+ were borderline significantly associated with increased risk of death compared to deferred lesions with negative concordance (iFR–/FFR–). FFR, fractional flow reserve; IFR, instantaneous wave-free ratio.

The network graph for the primary endpoint is presented in the **Supplementary Fig. 2**. Deferred lesions with FFR+/iFR– (OR: 1.67; 95% CI 0.68–4.11, *p* = 0.27; P-metric = 0.34) or FFR–/iFR+ (OR: 1.76, 95% CI 0.74–4.15, *p* = 0.2; P-metric 0.28) discordance were not significantly associated with increased risk for the composite clinical outcome compared to the reference group with negative concordance (Fig. 4). Heterogeneity was low with I^2^ = 0% (Q-test *p*-value = 0.94).

Regarding death, the group of deferred lesions with FFR+/iFR– did not have any events in the included studies. Therefore, this group was excluded from the analysis of death. Deferred lesions with FFR–/iFR+ discordance were significantly associated with an increased risk of death (OR: 3.19, 95% CI 1.004–10.16, *p* = 0.049; P-metric = 0.02) compared to the reference FFR–/iFR– group (Fig. 4). No test for heterogeneity was done for the outcome of death, based on the recommendations of the Cochrane collaboration, since a Mantel-Haenszel common effects model was used due to the fact that one group did not have any death events [25].

Regarding myocardial infarction in the network meta-analysis (**Supplementary Fig. 2**), compared to the reference group with negative concordance (FFR–/iFR–), deferred lesions with either FFR–/iFR+ (OR: 2.07, 95% CI 0.41–10.48, *p* = 0.38; P-metric = 0.27) or FFR+/iFR– (OR: 1.22, 95% CI 0.15–10.2, *p* = 0.85; P-metric = 0.54) were not significantly associated with increased odds (Fig. 4). No test for heterogeneity was done for the endpoint of myocardial infarction, based on the recommendations of Cochrane collaboration, since a Mantel-Haenszel common effects model was used due to the fact that there were only few events documented [25].

Lastly, the network analysis for revascularization (**Supplementary Fig. 2**; FFR–/iFR– group used as the reference), showed that deferred lesions with FFR+/iFR– were significantly associated with an increased risk of revascularization (OR: 3.24, 95% CI 1.3–8.05, *p* = 0.01; P-metric = 0.08), while deferred lesions with FFR–/iFR+ were not significantly associated with a greater risk of revascularization (OR: 1.52, 95% CI 0.46–5.05, *p* = 0.49; P-metric = 0.55) (Fig. 4). Heterogeneity was low with I^2^ = 0% (Q-test *p*-value = 0.89).

Lastly, by visually inspecting the funnel plots for each outcome, we can apparently conclude that there is no noteworthy publication bias (**Supplementary Figs. 9,12,15,18**), although the limitation of the small number of studies makes it difficult to assess publication bias accurately. Local inconsistency was assessed by visually inspecting the direct-indirect comparisons plots (**Supplementary Figs. 7,10,13,16**) and the SIDE tables (**Supplementary Figs. 8,11,14,17**). Regarding the composite primary endpoint and revascularization, it is essential to note that the local inconsistency analysis yielded extremely wide confidence intervals for both the primary composite endpoint and revascularization. Therefore, although the consistency assumption is met for these outcomes by inspecting the direct-indirect comparisons plot and the SIDE tables, the available data are too sparse to allow a reliable evaluation of inconsistency. Additionally, regarding death and myocardial infarction, there were no indirect comparisons; consequently, local inconsistency could not be assessed, and the network meta-analysis reduces to a pairwise direct synthesis for these two outcomes.

## 4. Discussion

We conducted a meta-analysis examining the clinical prognosis of deferred lesions with discordant FFR and iFR results compared to those with negative concordance, and the main findings are as follows: (1) Regarding the primary composite clinical outcome of deferred lesions, there was not a significant difference in adverse events between lesions with FFR/iFR discordance and lesion with negative concordant results, albeit with a numerically higher event rate for the former group; (2) although the primary analysis did not show a significant difference in death between lesions with discordant FFR/iFR classification and concordant negative results, the secondary analysis demonstrated that deferring lesions specifically with FFR–/iFR+ results was associated with an increased risk of death compared to deferring lesions with negative concordance; (3) there was a trend for a higher rate of revascularization in deferred lesions with FFR/iFR discordance compared to deferred lesions with negative concordance, and the secondary analysis demonstrated that deferred lesions specifically with FFR+/iFR– were significantly associated with an increased risk of revascularization compared to deferred lesions with negative concordance.

The previously published observational studies overall show similar results with numerically higher event rates (in the groups with discordant FFR/iFR classification compared to the negative concordant ones) without reaching statistical significance [17, 30, 31, 32]. Our meta-analysis takes advantage of the synthesis of data further highlighting the numerical differences in event rates. Additionally, our network meta-analysis demonstrated that deferred lesions with discordant FFR+/iFR– results are significantly associated with an elevated risk of revascularization compared to the concordant FFR–/iFR– results. This is partly in accordance with the significantly higher rate of ischemia-driven revascularization and VOCO in cases with discordant FFR/NHPR classification (compared to the group with negative concordant results) observed in the study by Lee JM *et al*. [18] with the longer clinical follow-up.

Several clinical and angiographic characteristics are associated with the presence of discordance between FFR/iFR results. Atrial fibrillation and insulin-treated diabetes mellitus have been described as predictors of FFR/iFR discordance [10]. Additionally, an angiographically diffuse disease has been associated with FFR–/iFR+ discordance, while a focal disease has been linked to FFR+/iFR– discordance [11]. Lesions in the LAD are also related to discordant FFR/iFR classification [11]. Other predictors of discordant results include chronic kidney disease and age [14]. Notably, the ADVISE II study demonstrated that the hyperaemic response is age-dependent; thus, FFR values tend to increase as age increases, while iFR values remain unchanged [36]. This finding needs to be taken into consideration when evaluating borderline lesions with discordant values in the elderly before deciding to either defer or proceed to revascularization. Another critical factor to consider is that chronic kidney disease, especially in the lower eGFR ranges, is related to an attenuated hyperaemic response, a finding more pronounced in non-LAD lesions [16]. Therefore, the decision to treat such lesions must be taken cautiously. Additionally, diastolic dysfunction, evaluated by the E/E’ ratio, is another factor related to discordant FFR/iFR results and should be taken into consideration when conducting physiology measurements [37].

Many studies have investigated the patterns of lesions with discordant and concordant FFR/iFR results, focusing on physiology, ischemia, and vascular parameters. When the physiologic characteristics of lesions with discordant FFR/iFR results were studied, it was shown that lesions with FFR–/iFR+ had similar results regarding CFR, resistive reserve ratio (RRR), and the index of microcirculatory resistance (IMR) to those with positive concordance (FFR+/iFR+) [17, 38], possibly indicating a similarly unfavourable physiological pattern between these groups of lesions. Such a finding may be the reason for the higher risk of death associated with deferring lesions with FFR–/iFR+ results. Furthermore, investigation of lesions with concordant and discordant FFR/iFR and their patterns in ^13^N-ammonia PET has demonstrated that lesions with discordant results had similar unfavourable PET-derived parameters to those with concordant abnormal results [39]. This finding supports that discordant lesions may have a similar adverse pattern of behavior to lesions with concordant abnormal results (i.e., concordant positive values).

Additionally, lesions with abnormal FFR and normal NHPRs have been associated with atherosclerotic systemic vascular damage as assessed by the ankle-brachial pressure index (a marker of arterial stenosis) and brachial-ankle pulse wave velocity (a marker of arterial stiffness) [40], while endothelial dysfunction, as evaluated by the reactive hyperemia index, has also been associated with discordance between FFR/iFR [41]. Of note, although both FFR and iFR abnormal values are associated with a higher risk of high-risk plaque characteristics, FFR has a more pronounced discriminatory capacity [42]. This finding may explain why lesions with FFR+/iFR– results were associated with a greater risk of revascularization compared to lesions with FFR–/iFR+ or FFR–/iFR– results. A myocardial perfusion scintigraphy (MPS) study demonstrated that lesions with discordant results of FFR and NHPR have lower MPS-driven ischemia than lesions with concordant abnormal values, but greater ischemia than lesions with concordant normal values [43]. Therefore, such lesions may be a target for revascularization, even though the decision to proceed to PCI or defer is not straightforward.

There are limited literature data regarding studies that directly compare a deferral or revascularization approach for lesions with discordant FFR/iFR results and evaluate the comparative efficacy and safety of these approaches. The study by Lee *et al*. [18] examined the long-term safety of deferred lesions with either negative concordant or discordant results, as well as revascularized lesions, providing a first indirect insight. The study demonstrated that deferred lesions with negative concordant results were associated with a significantly lower risk of adverse clinical outcomes [18]. In contrast, revascularized lesions and deferred lesions with discordant results were associated with an increased risk of such outcomes [18]. However, it showed that despite a similar risk profile of lesions with at least one abnormal result of physiology indices, a deferral approach may be comparable to a revascularization approach.

Another clinically important issue is whether iFR-guided management is indeed non-inferior to FFR-guided management. According to the DEFINE-FLAIR [5] and iFR-SWEDEHEART [6] trials, iFR-guided management was shown to be non-inferior to FFR-guided management based on the 12-month analysis, albeit a pooled meta-analysis of these two trials regarding death and myocardial infarction showed a statistical trend for higher rate of death and myocardial infarction in the iFR-guided management group [44]. When the 5-year results of the two trials were published [45, 46], a repeat pooled meta-analysis which demonstrated that iFR-guided management was associated with a significantly higher rate of death compared to FFR-guided management [47]. Another pooled analysis of the 5-year results of DEFINE-FLAIR and iFR-SWEDEHEART by Eftekhari *et al*. [48] demonstrated that iFR-guided revascularization was associated with increased risk of all-cause mortality and MACE compared to FFR-guided management. These findings are of paramount importance and require further evaluation through dedicated studies. Large and well-designed trials are necessary to investigate further whether a revascularization or deferral approach for lesions with discordant FFR/iFR classification is superior in terms of safety and efficacy, and to shed light on this controversial topic.

### Limitations

The lack of any randomized trials designed to answer the research question is a weakness of this meta-analysis, given that all the evidence used in our analysis is based on observational studies. Furthermore, the included studies were not designed prospectively to answer the clinical issue in question, and they may have been underpowered to detect differences between deferred lesions with concordant and discordant FFR/iFR results. Another limitation of our meta-analysis is the rather small number of included studies. However, only a small number of studies including focused FFR and iFR assessment in deferred lesions are available in the literature. Although the rather small number of studies may limit the statistical power, our meta-analytic results enhance the findings of the original studies and highlight clinically important differences. Lastly, there was variability regarding the definition of the outcomes among the different studies included in the analysis, in particular the outcome of death, with some studies reporting all-cause death and others cardiac death; this issue may have diluted the ability to detect cardiac-specific death. However, the heterogeneity for all outcomes among the studies included in the meta-analysis was low for both the primary and secondary analyses.

## 5. Conclusions

Our meta-analytic data indicate that FFR/iFR discordance in deferred lesions may not be benign. Deferred lesions with discordant results of FFR/iFR are not significantly associated with an increased risk of the composite clinical outcome, death, myocardial infarction, and revascularization compared to deferred lesions with negative concordant results, albeit presenting numerically higher event rates. Deferred lesions with FFR+/iFR– are significantly associated with an increased risk of revascularization compared to lesions with negative concordant results. Conversely, deferred lesions with FFR–/iFR+ seem to be associated with an increased risk of death. Dedicated and large studies are needed to provide definitive evidence on whether deferring lesions with discordant FFR/iFR results is associated with an increased risk of clinical outcomes compared with deferred lesions with negative concordance, while randomized trials with long-term follow-up that directly compare deferring or proceeding to PCI for lesions with discordant iFR/FFR results are necessary to shed light on this topic and potentially improve clinical decision making.

## Availability of Data and Materials

The datasets used during the current study are available from the corresponding author on reasonable request.

## Supplementary Material

Supplementary material associated with this article can be found, in the online version, at https://doi.org/10.31083/RCM44868.
