# Supplementary material for: Clinical Outcomes Following Discordance Between Fractional Flow Reserve and Instantaneous Wave-Free Ratio in Deferred Coronary Lesions: A Systematic Review and Meta-Analysis
Source: Rev Cardiovasc Med. 2025 Nov 21;26(11):44868. doi: 10.31083/RCM44868 (PMC12680999; doi:10.31083/RCM44868)

**Clinical outcomes following discordance between fractional flow reserve and instantaneous wave-free ratio in deferred coronary lesions: a systematic review and meta-analysis**

**SUPPLEMENTARY MATERIAL**

### Supplementary Fig. 1. Forest plot of leave-one-out analysis


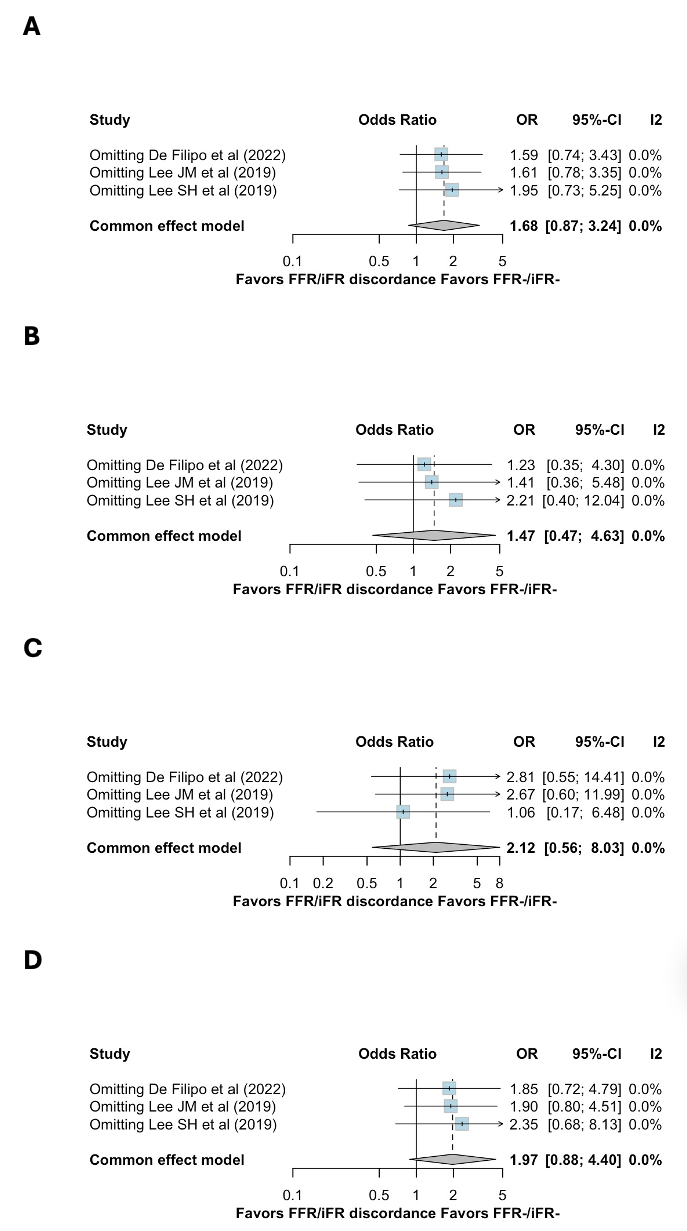


Supplementary Fig. 1. Forest plots for all endpoints of the leave-one-out analysis. A. Composite clinical outcome. B. Death. C. Myocardial infarction. D. Revascularization.

### Supplementary Fig. 2. Funnel plot for publication bias for the composite primary endpoint in the primary analysis


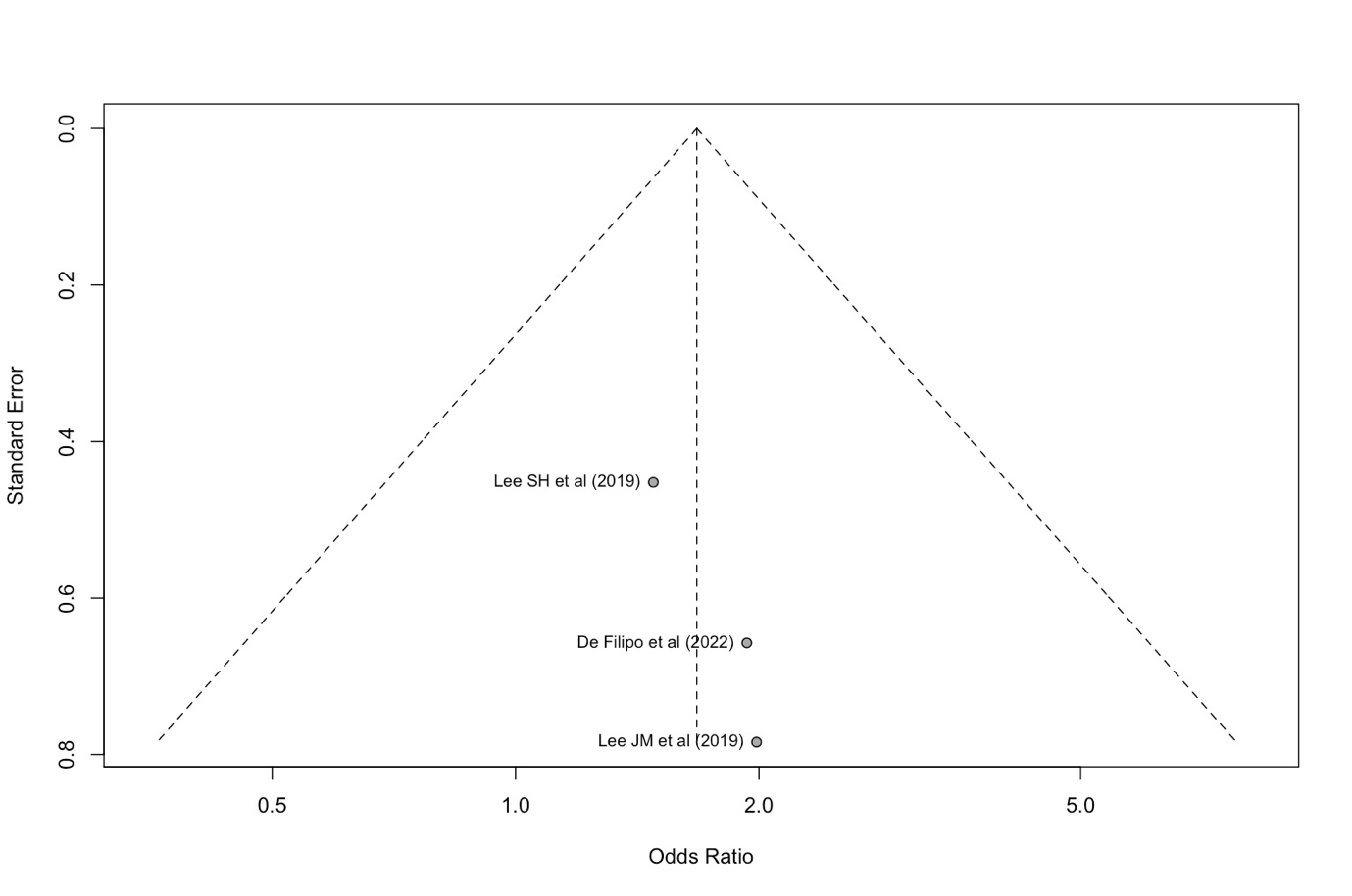


### Supplementary Fig. 3. Funnel plot for publication bias for death in the primary analysis


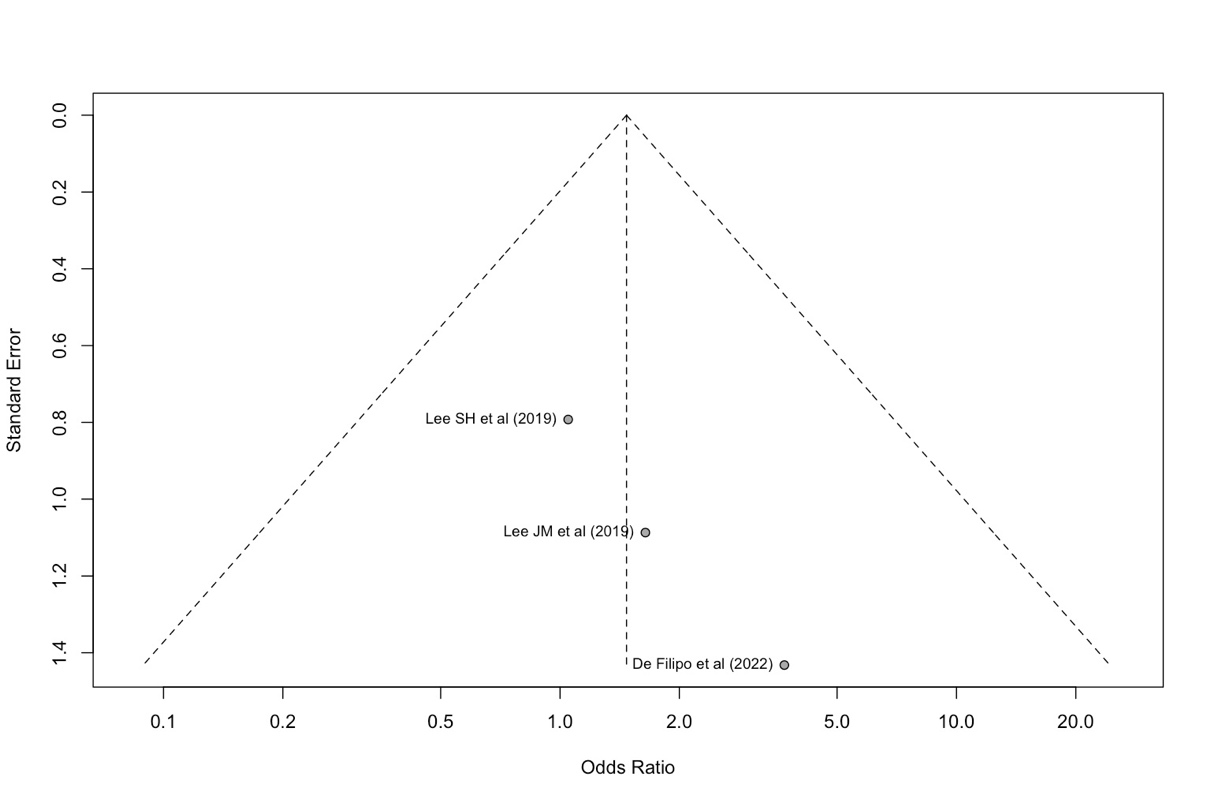


### Supplementary Fig. 4. Funnel plot for publication bias for myocardial infarction in the primary analysis


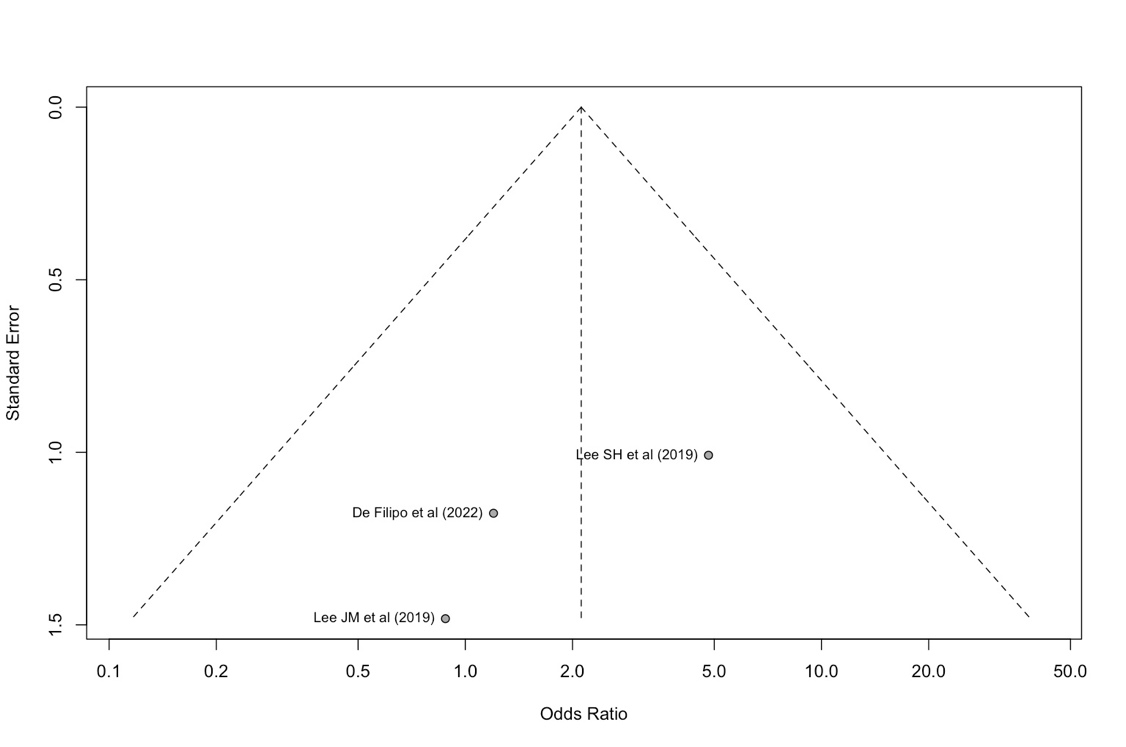


### Supplementary Fig. 5. Funnel plot for publication bias for revascularization in the primary analysis


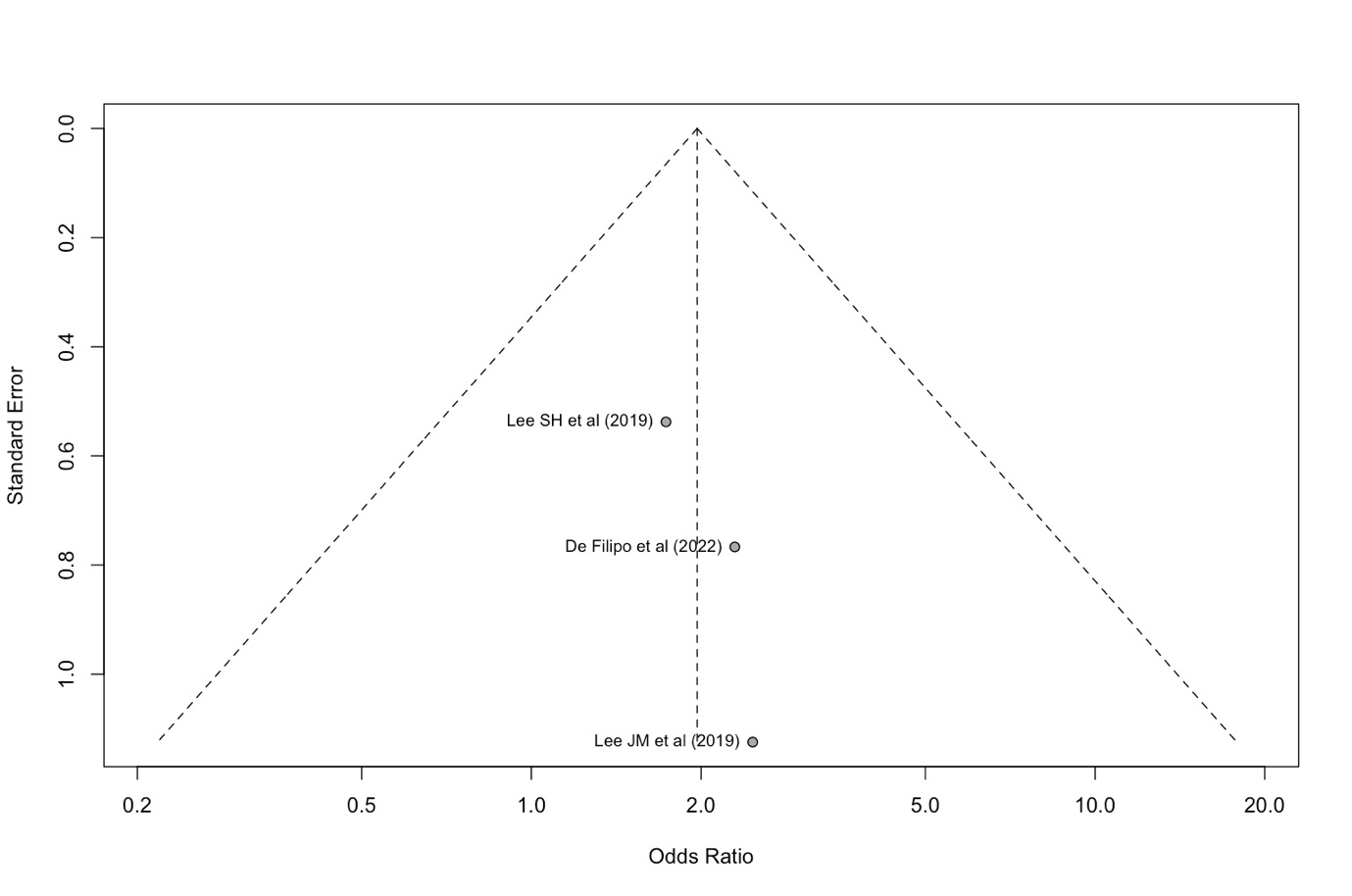


### Supplementary Fig. 6. Network graph for the composite primary endpoint, myocardial infarction and revascularization


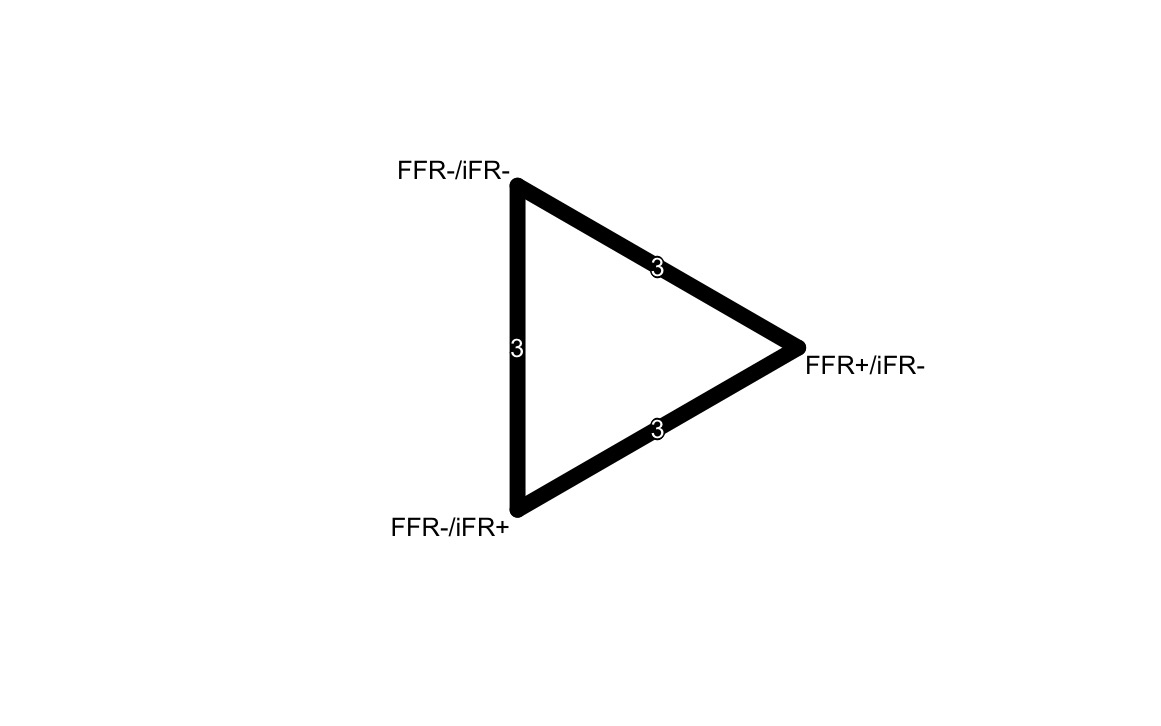


### Supplementary Fig. 7. Forest plot of the direct and indirect evidence for the individual comparisons for the composite primary endpoint


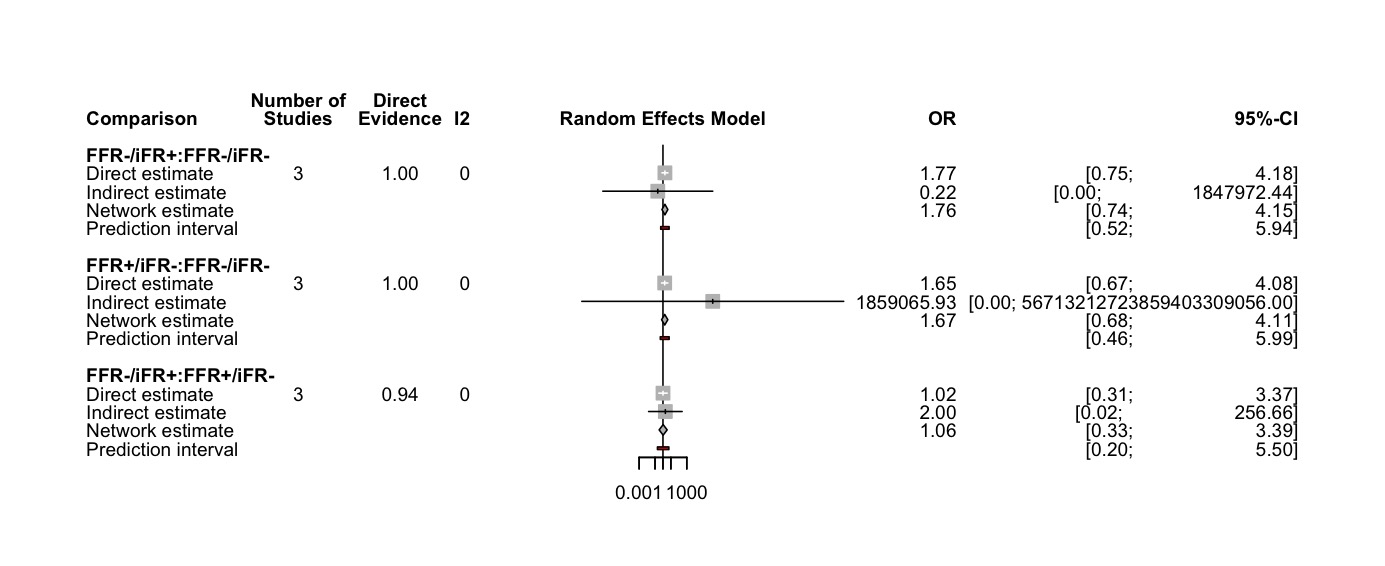


### Supplementary Fig. 8. SIDE (separating indirect from direct evidence) table for the composite primary endpoint

| **Comparison** | **Number of studies with direct evidence** | **Direct evidence proportion** | **nma** | **OR derived from direct evidence** | **OR derived from indirect evidence** | **Ratio of ratio (RoR)** | **z-value** | **p-value** |
| --- | --- | --- | --- | --- | --- | --- | --- | --- |
| FFR-/iFR+:FFR-/iFR- | 3 | 1.00 | 1.758 | 1.769 | 0.223 | 7.936 | 0.25 | 0.7991 |
| FFR+/iFR-:FFR-/iFR- | 3 | 1.00 | 1.665 | 1.652 | 1,859,065.934 | 0.000 | -0.72 | 0.4720 |
| FFR-/iFR+:FFR+/iFR- | 3 | 0.94 | 1.056 | 1.015 | 2.004 | 0.507 | -0.27 | 0.7898 |

### Supplementary Fig. 9. Funnel plot for publication bias for the composite primary endpoint


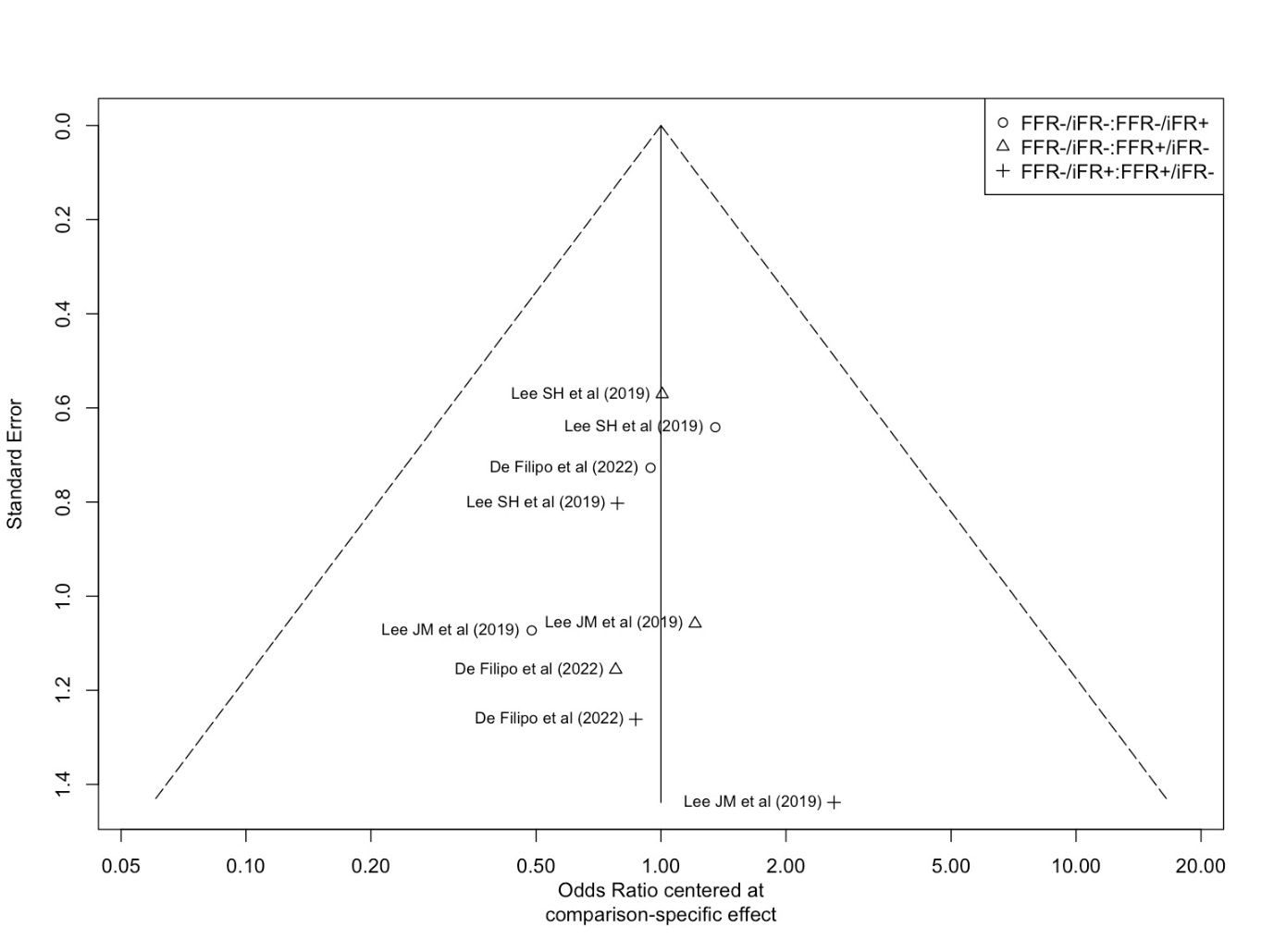


### Supplementary Fig. 10. Forest plot of the direct and indirect evidence for the individual comparisons for death


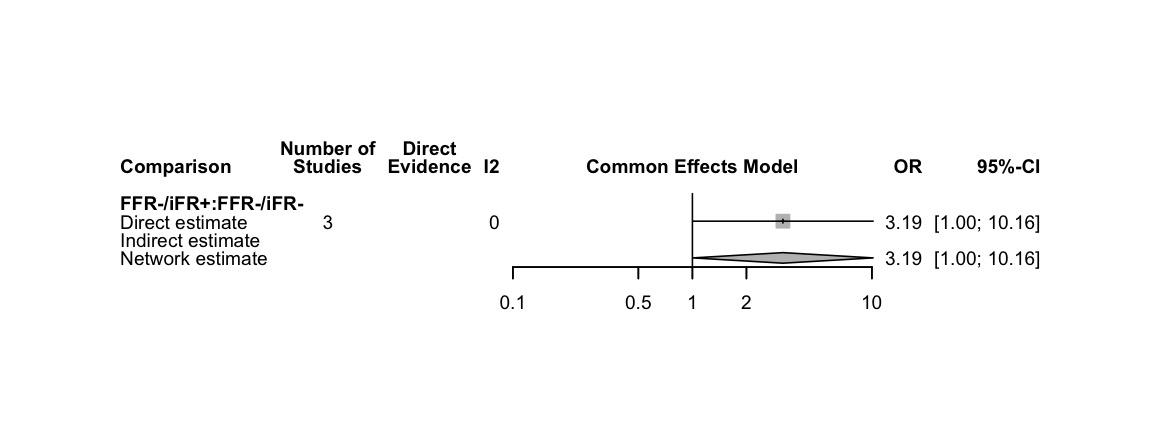


### Supplementary Fig. 11. SIDE (separating indirect from direct evidence) table for death

| **Comparison** | **Number of studies with direct evidence** | **Direct evidence proportion** | **nma** | **OR derived from direct evidence** | **OR derived from indirect evidence** | **Ratio of ratio (RoR)** | **z-value** | **p-value** |
| --- | --- | --- | --- | --- | --- | --- | --- | --- |
| FFR-/iFR+:FFR-/iFR- | 3 | - | 3.193 | 3.193 | - | - | - | - |

### Supplementary Fig. 12. Funnel plot for publication bias for death


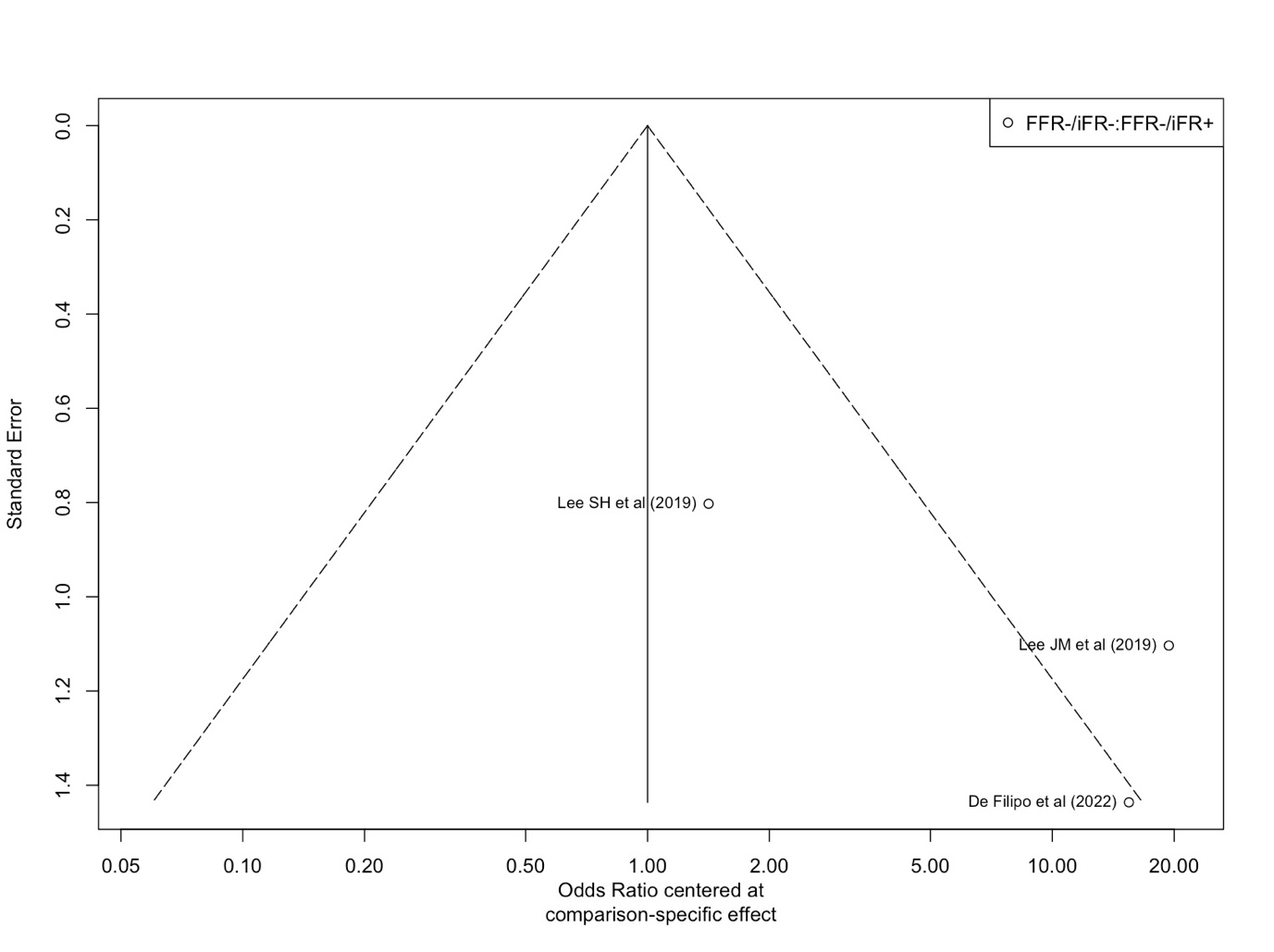


### Supplementary Fig. 13. Forest plot of the direct and indirect evidence for the individual comparisons for myocardial infarction


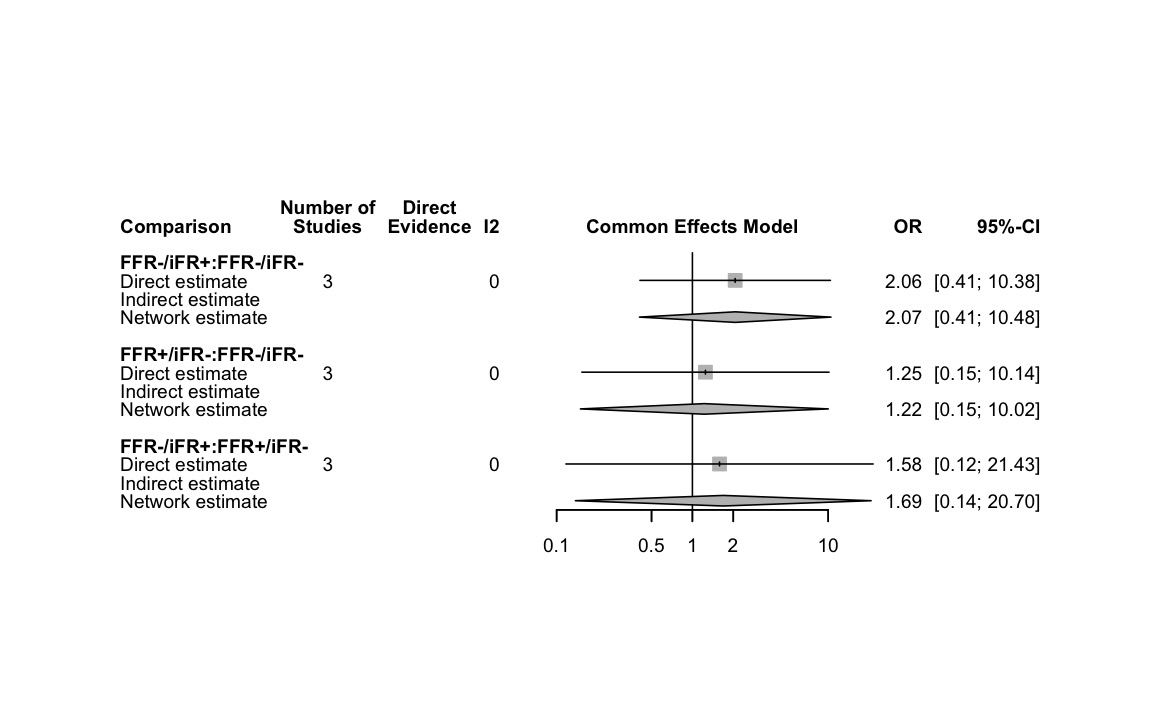


### Supplementary Fig. 14. SIDE (separating indirect from direct evidence) table for myocardial infarction

| **Comparison** | **Number of studies with direct evidence** | **Direct evidence proportion** | **nma** | **OR derived from direct evidence** | **OR derived from indirect evidence** | **Ratio of ratio (RoR)** | **z-value** | **p-value** |
| --- | --- | --- | --- | --- | --- | --- | --- | --- |
| FFR-/iFR+:FFR-/iFR- | 3 | - | 2.066 | 2.065 | - | - | - | - |
| FFR+/iFR-:FFR-/iFR- | 3 | - | 1.225 | 1.247 | - | - | - | - |
| FFR-/iFR+:FFR+/iFR- | 3 | - | 1.687 | 1.584 | - | - | - | - |

### Supplementary Fig. 15. Funnel plot for publication bias for myocardial infarction


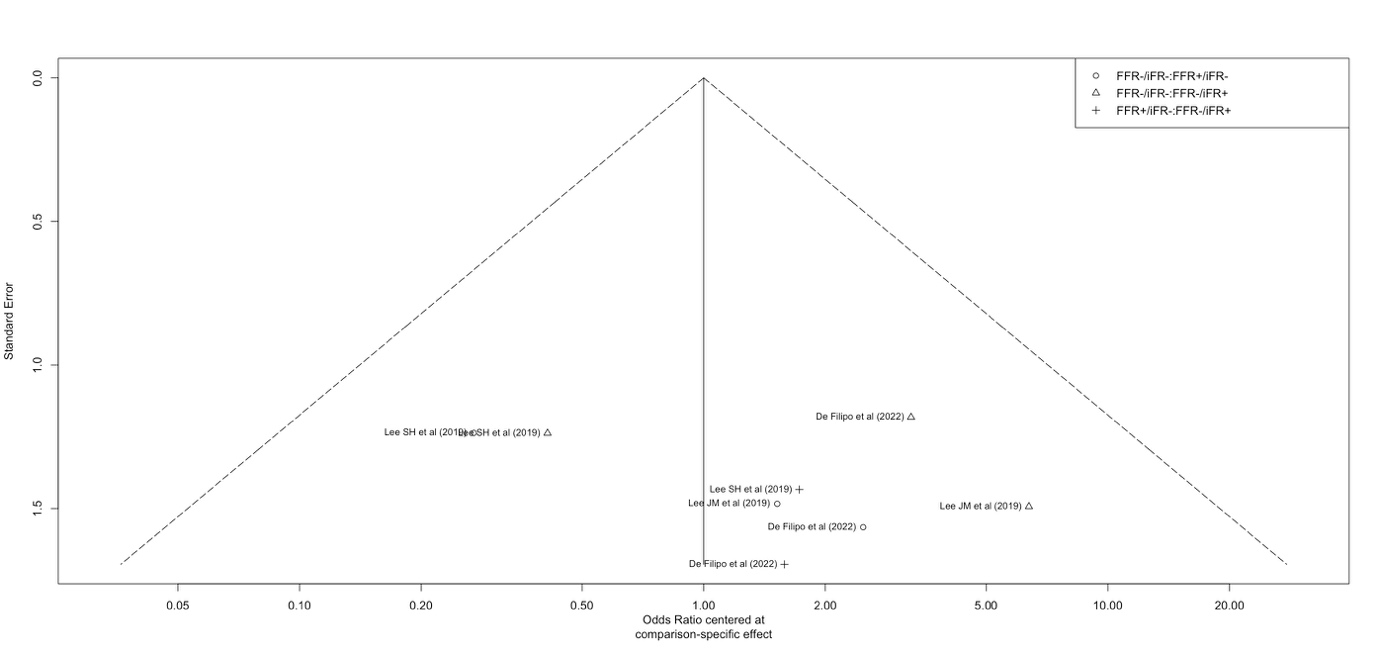


### Supplementary Fig. 16. Forest plot of the direct and indirect evidence for the individual comparisons for revascularization


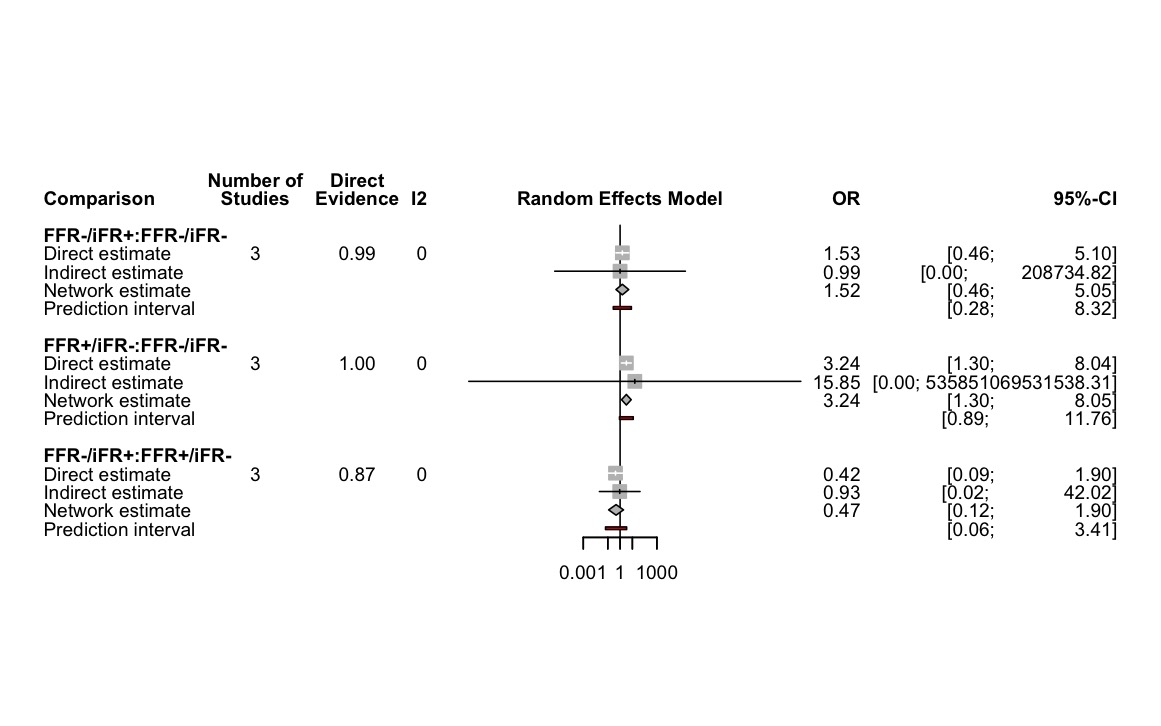


### Supplementary Fig. 17. SIDE (separating indirect from direct evidence) table for revascularization

| **Comparison** | **Number of studies with direct evidence** | **Direct evidence proportion** | **nma** | **OR derived from direct evidence** | **OR derived from indirect evidence** | **Ratio of ratio (RoR)** | **z-value** | **p-value** |
| --- | --- | --- | --- | --- | --- | --- | --- | --- |
| FFR-/iFR+:FFR-/iFR- | 3 | 0.99 | 1.524 | 1.531 | 0.992 | 1.542 | 0.07 | 0.9450 |
| FFR+/iFR-:FFR-/iFR- | 3 | 1.00 | 3.240 | 3.236 | 15.850 | 0.204 | -0.10 | 0.9204 |
| FFR-/iFR+:FFR+/iFR- | 3 | 0.87 | 0.470 | 0.423 | 0.931 | 0.454 | -0.38 | 0.7057 |

### Supplementary Fig. 18. Funnel plot for publication bias for revascularization


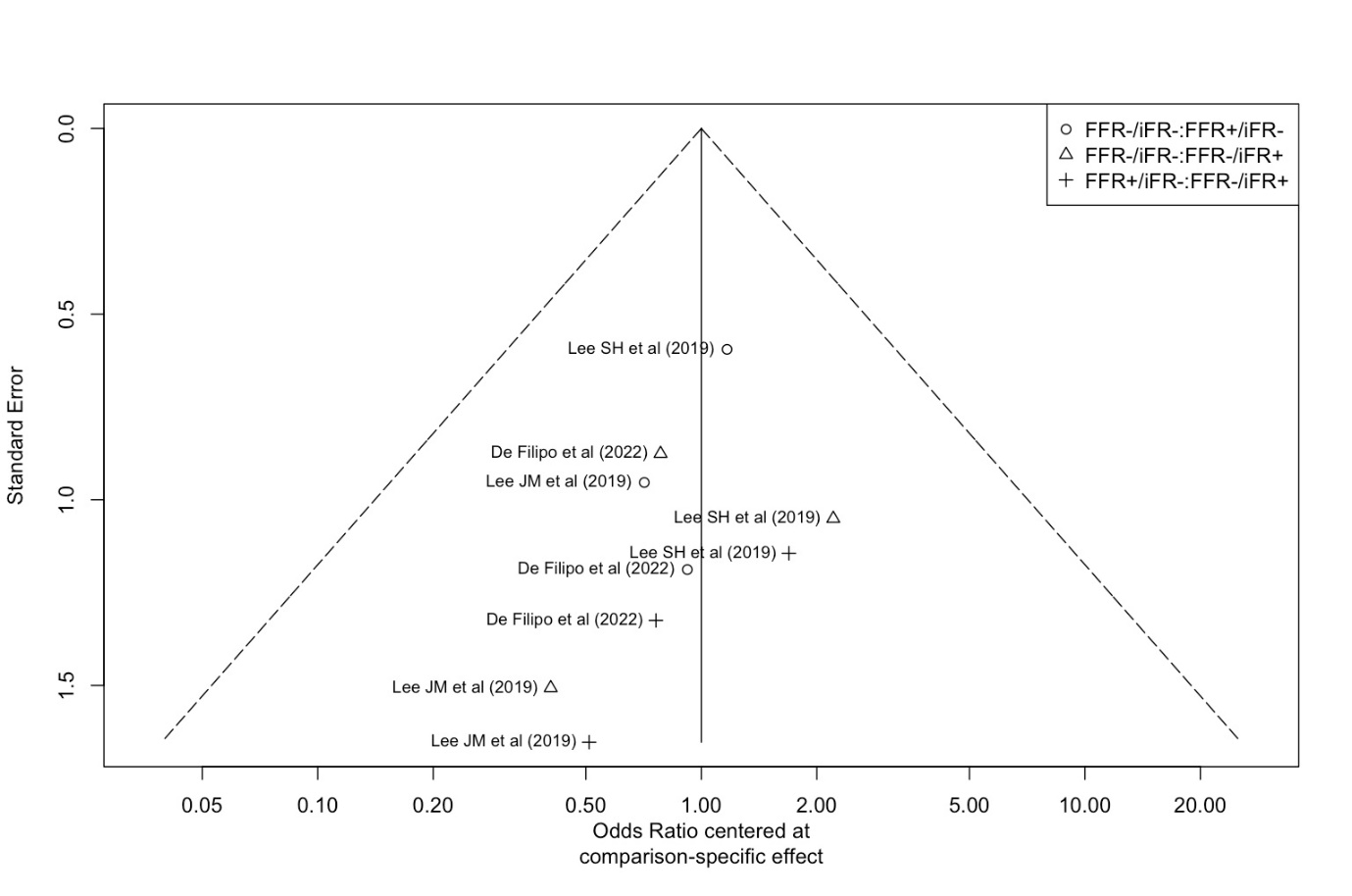

Supplement: Supplementary file 1 [file 2153-8174-26-11-44868-s1.zip › Supplementary Material.docx]
